# Effect of Folded Structures on Interfacial Solar-Driven Seawater Desalination

**DOI:** 10.3390/membranes15050134

**Published:** 2025-05-01

**Authors:** Shufang Zhu, Yuke Niu, Xu Yan

**Affiliations:** 1 Industrial Research Institute of Nonwovens &Technical Textiles, Shandong Center for Engineered Nonwovens, College of Textiles & Clothing, Qingdao University, Qingdao 266071, China; 2020020729@qdu.edu.cn (S.Z.); niuyuke@qdu.edu.cn (Y.N.); 2 Shandong Yuma Sun-Shading Technology Corp, Ltd., Shouguang 262702, China; 3 State Key Laboratory of Bio-Fibers and Eco-Textiles, Qingdao University, Qingdao 266071, China

**Keywords:** interfacial solar evaporator, folded structure, recycled PET, electronspinning, desalination

## Abstract

Currently, solar-driven interface evaporation for seawater desalination is believed to be an effective way to overcome freshwater shortage. To improve the efficiency of solar-driven interfacial evaporators, designing the evaporator’s structure is essential. In this study, we proposed a folded structure solar-driven interfacial evaporator with electrospun recycled PET/carbon nanotube fibrous membranes. The as-spun membranes were folded into 4, 8, and 16 petals. The results suggested that F@8 (fold with eight petals) had the best solar-driven evaporation performance, with a photothermal conversion efficiency of 90.59% and an evaporation rate of 1.31 kg·m^−2^·h^−1^, due to its lower light projection area and greater light absorption. The evaporation performance remained stable after 10 cycles. In addition, the concentration of ions in the freshwater collected after desalination was 2~3 orders of magnitude lower than that before desalination. These results indicate that a properly designed folded structure can effectively enhance evaporators through changing the light projection area and absorption. This approach might provide an effective way to optimize the structure of interfacial solar-driven evaporators.

## 1. Introduction

Freshwater shortage is one of the serious challenges facing the world [1]. In order to address this challenge, researchers are constantly exploring and developing new water resource utilization technologies, among which seawater desalination technology has become one of the effective ways to solve freshwater shortage [2]. Traditional seawater desalination technologies mainly include distillation [3], reverse osmosis [4], electrolysis [5], and electrodialysis [6]. These technologies have disadvantages such as a high energy consumption, high cost, high brine water, and CO_2_ pollution to the environment [7]. In recent years, solar-driven interface evaporation technology has attracted much attention for its clean and renewable advantages [8].

The main influencing factors of solar-driven interface evaporation technology include its light absorption capacity, water transport capacity, salt resistance, and thermal management capacity [9]. Among these, light absorption capacity plays a crucial role [10]. This is mainly determined by the photothermal materials and the structural design of the evaporator [11]. Specifically, different types of materials have varying abilities to absorb light, such as metal materials [12], semiconductor materials [13], carbon-based materials [14], and Mxene nanocomposite material [15].

Meanwhile, the structural design of the evaporator plays an important role in its light absorption performance [16]. Wang et al. discovered that despite the identical mass and volume of the materials used, the evaporation rate was greatly increased by a straightforward structural change from a 2D to a 3D evaporator [17]. This might have been due to increases in light absorption and the evaporation surface area, which increased the contact area between light and the material, thereby improving the light absorption efficiency [18,19]. Liang et al. developed an arched solar evaporator with an all-round light capture capability, significantly increasing light utilization efficiency [20]. Li et al. developed an M-shaped solar evaporator with an optimized optical path, significantly reducing light loss [21]. Zhao et al. designed a three-position spiral solar evaporator with the ability to provide uniform illumination, significantly increasing solar energy utilization [22]. Lim et al. developed a 3D rice-paddy-shaped solar evaporator with an internal radiation capability, significantly increasing the propagation of light [23]. Jia et al. designed a three-dimensional water evaporator for carbonized silkworm cocoons with the ability to guide light, significantly increasing the contact time of light [24]. Sun et al. developed a 3D mirror-assisted concave pyramid-shaped solar hot water evaporation system with the ability to concentrate light, significantly reducing light loss [25]. Comprehensively, the designed structures mainly were topological ones, with different kinds of special geometrically shaped macrostructures [26,27]. Among these structures, folding ones have attracted a lot of interest due to their ease of construction [28,29,30,31,32]. However, how these folding structures affect solar-driven evaporation is still an unanswered question.

In this study, we designed a low-cost folded 3D evaporator with carbon nanotubes and electrospun recycled PET fibrous membranes. SEM and water contact angles were used to analyze the electrospun membranes’ morphology and surface wettability. Moreover, the folded structures were designed with different numbers of folded petals, 0, 4, 8, and 16, respectively. The photothermal seawater desalination performances of these evaporators were tested under one solar intensity (1 kw/m^2^).

## 2. Materials and Methods

### 2.1. Materials

Plastic bottles of Laoshan Spring Water were used to produce recycled PET (rPET) to reduce the cost of the evaporator [32,33]. Tanfeng Tech.Inc supplied the multi-walled carbon nanotubes (inner diameter of 3–5 nm, outside diameter of 8–15 nm, and specific surface area of 250 m^2^/g), which were utilized as photothermal particles. Trifluoroacetic acid (AR, ≥99.5%) was employed as a solvent after being acquired from Shanghai Aladdin Biochemical Technology Co., Ltd. (Shanghai, China). Shanghai Aladdin Biochemical Technology Co., Ltd. (Shanghai, China) was the supplier of NaOH (AR, ≥96.0%) and H_2_SO_4_ (AR, 95.0~98.0%). Polystyrene (PS) foams were recycled from the express station in Qingdao University and washed with deionized water. Air-laid papers (XWI80024) were purchased from the Xinweike flagship store on Tmall. Seawater was obtained from China’s Yellow Sea, Qingdao, and left to stand for 24 h.

### 2.2. Fabrication of Photothermal CNTs@rPET Membranes

**Figure 1:**
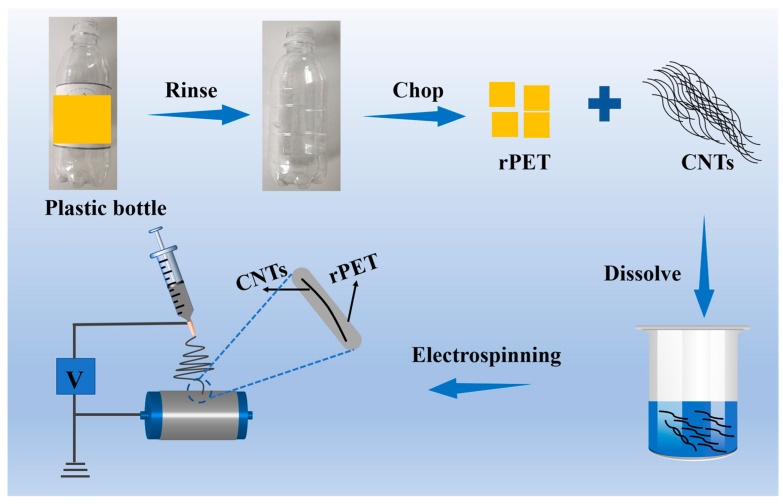
The preparation process of rPET@CNTs-P fiber membrane.

Figure 1 shows the electrospinning and rPET solution preparation procedures. The water bottle used was first cleaned with deionized water before drying in open air. The second step involved cutting the dry bottle into 1 × 1 cm^2^ pieces, which were then dissolved in trifluoroacetic acid with a concentration of 10 wt% under magnetic stirring for 8 h and then left to stand for 24 h. Based on previous research, we dispersed CNTs of different concentrations into the above solution using ultrasound for 2 h, leading to varying CNTs to rPET ratios (CNTs@rPET, 0.5, 2.0, 3.5, and 5.0 wt%) [32]. Electrospinning apparatus (JDF05, Na Yi Instrument Co., Ltd., Changsha, China) was equipped with the prepared solution after it had been put into a 5 mL syringe with a 21# metal flat needle (inner diameter of 0.51 mm). The electrospinning process was conducted under climate-controlled (air conditioning) lab conditions with a temperature and humidity of about 22 ± 3 °C and 50 ± 5% RH. The spinning parameters were set as follows: an applied voltage of 13~14 kV, a receiving distance of 15 cm, a feeding rate of 0.3 mL/h, a collect roller speed of 1000 r/min, and needle reciprocating motion at 5 cm/min. After 2 h, we obtained a film thickness of about 200–250 μm. This thickness ensured that the based evaporator worked well. An air-laid paper (20 cm width and 30 cm length) was fixed onto the collect roller as the receiving substrate, and the as-spun films were named CNTs@rPET-P, with P as the air-laid paper [32]. Each membrane was electrospun for 2 h.

### 2.3. Preparation of Folded Structure 3D Evaporator

**Figure 2:**
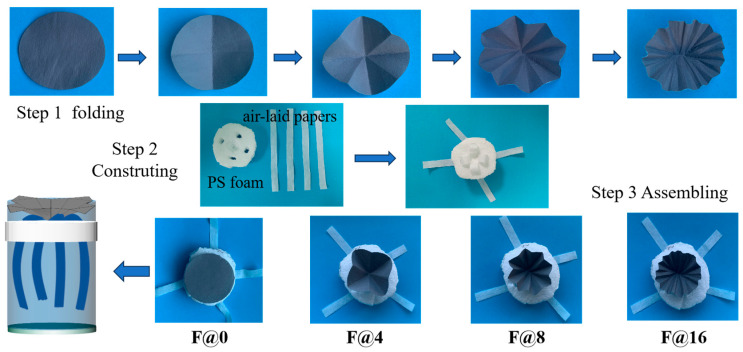
The assembly structure diagram of F@N.

It was discovered that a folding structure design can significantly improve the efficiency of light absorption through the multiple reflection mechanism of light [28,29,30,31,32,34]. Therefore, in order to improve the light absorption rate, the CNTs@rPET-P film (with a radius of 2.5 cm) was folded into a 3D light absorber, named the F@N evaporator, where N is the number of folded petals, 0, 4, 8, and 16, respectively. As shown in Figure 2, there were three steps for preparing the F@N evaporators. Firstly, the as-spun CNTs@rPET-P membranes were folded into a petal structure with 0, 4, 8, and 16 petals. Compared to other folding structures, ths petal structure is not only easy to operate, but also compact and portable. This folding structure can cause the incident light to undergo multiple refractions and reflections before reaching the bottom surface, greatly reducing the amount of light directly reflected back to the outside world, allowing for more light to be absorbed by the absorber [32,34]. Secondly, the recycled PS foam was cut into a circular shape with a diameter of 6 cm and a thickness of 2.5 cm, and four holes and a concave pit were dug through (1 cm deep), displayed as step 2 in Figure 2. The four holes were inserted into four air-laid paper strips (10 cm length and 1 cm width), respectively, and a square pit was left in the center (1.5 cm wide and 2 cm deep). Thirdly, the folded CNTs@rPET-P membranes in step 1 were assembled and fixed into the square pit on the PS foam, as shown in step 3 in Figure 2. The whole F@N evaporators could then be achieved with the folded CNTs@rPET-P membrane as a photothermal layer, the PS foam as a support and heat insulation material, and the air-laid paper as a seawater transport channel. The F@N evaporators could then be placed onto the seawater surface and absorb sunlight to generate steam.

### 2.4. Characterization

Scanning electron microscopy (SEM, S4800, Hitachi, Japan), field-emission scanning electron microscopy (FE-SEM, Regulus 8100, Hitachi, Japan), and transmission electron microscopy (TEM, 2100 Plus, Japan Electronics Co., Ltd., Amagasaki, Japan) were used to examine the morphology and microstructure of the electrospun CNTs@rPET-P membranes. A UV–Vis NIR diffuse reflectance spectrophotometer (PE lambda 750, PerkinElmer Instruments Co., Ltd., Waltham, MA, USA) was used to characterize the light absorbance of the as-spun membranes. The surface wettability of the as-spun membranes was characterized using a contact angle analyzer (JY-PH, Chengde Jinhe Instrument Manufacturing Co., Ltd., Chengde, China) at 3 randomly selected sites.

### 2.5. Photothermal Evaporation Experiments

The photothermal evaporation performance was examined under a solar simulator xenon lamp (CEL-PE300L-3A xenon lamp, Beijing Zhongjiao Jinyuan Technology Co., Ltd., Beijing, China) with a light intensity of 1 kw/m^2^, which was directly above the prepared evaporator at a distance of about 30 cm. The experiments were processed in an air-conditioned controlled lab with a relatively stable temperature and humidity of about 22 ± 3 °C and 50 ± 5% RH. An optical power densitometer (CEL-NP2000-2A, Beijing Zhongjiao Jinyuan Technology Co., Ltd., Beijing, China) was used to determine the optical power density. An infrared camera (FLIR, tis 20+) was applied to record the surface temperature of the evaporator in real time (10 s) and a precision electronic balance (JC-JA11003, Shanghai Yueping Science Instrument Co., Ltd., Shanghai, China) was used to measure the mass change in seawater during steam generation in real time. During the photothermal evaporation experiments, the evaporator and a beaker of seawater were placed on the electronic balance, and then the seawater mass change was recorded 5 times. Finally, we used ICP-OES/MS (PerkinElmer 8300, Shimadzu, Japan) to determine the ion concentration in the seawater before and after desalination.

## 3. Results and Discussion

### 3.1. Morphology of the As-Spun CNTs@rPET Membranes

**Figure 3:**
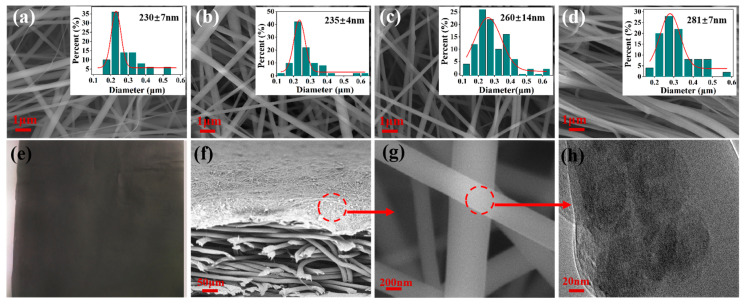
(**a**–**d**) SEM images and fiber diameter distribution of the as-spun CNTs@rPET membranes with carbon nanotubes concentrations of 0.5, 2, 3.5. and 5 wt%, respectively. (**e**) The real image of the CNTs@rPET-P with 5 wt% CNTs. (**f**) The CNTs@rPET-P membrane’s cross section SEM picture. (**g**) The enlarged SEM image of CNTs@rPET. (**h**) The TEM picture of CNTs@rPET fiber.

Figure 3a–d show the micro morphology of the as-spun CNTs@rPET membranes doped with carbon nanotube concentrations of 0.5, 2.0, 3.5, and 5.0 weight percent. The electrospun CNTs@rPET fibers were found to have a smooth surface and thinner diameters of around 230–280 nm (by counting 100 fibers for each sample). As seen in the enlarged images in Figure 3a–d, the addition of carbon black resulted in a rise in fiber diameters from 230 ± 7 nm to 280 ± 7 nm along with the number of CNTs. This increased fiber diameter can be attributed to the increase in viscosity with an increasing carbon nanotube concentration. When the viscosity of the solution increased, the interaction force between polymer segments improved, resulting in a decrease in the mobility of the segments [35]. This made it more difficult for polymer segments to be stretched and refined under the action of electric field force. Figure 3e shows that the prepared electrospun CNTs@rPET membrane was black overall because of the presence of carbon nanotubes. Figure 3f shows that the as-spun CNTs@rPET membrane was located onto an air-laid paper, then formed a double-layer structure. As shown in Figure 3g, the electrospun membrane was composed of many fibers with uniform diameters, which were randomly interwoven to form pores that facilitated the escape of water vapor [36]. As seen in Figure 3h, CNT particles were evenly wrapped in rPET from the TEM image, which ensured that the CNTs would not detach due to external mechanical forces, the folding process, or adverse environmental influences, thereby enhancing the stability of their photothermal performance.

### 3.2. Wettability and Stability of CNTs@rPET-P Membrane

**Figure 4:**
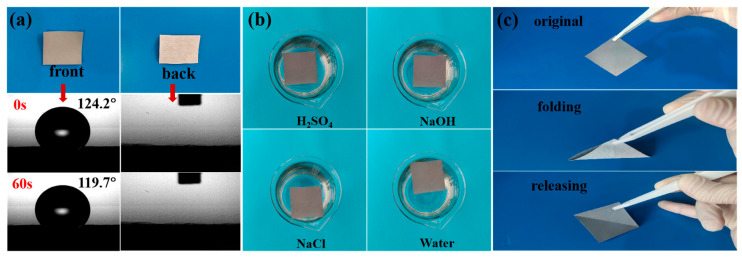
(**a**) The water drop onto the front and back sides of the CNTs@rPET-P membranes. (**b**) The stability test of photothermal film of CNTs@rPET-P in different solutions. (**c**) The flexibility test of CNTs@rPET-P.

One key factor in ensuring quick water evaporation was hydrophilicity [37]. As shown in Figure 4a, the front side of the CNTs@rPET-P membrane exhibited an excellent hydrophobicity, with water contact angles of about 120°, which was beneficial for preventing salt deposition. Meanwhile, the back side also exhibited an excellent hydrophilicity, ensuring that water could be continuously transported from the bottom to the top. Like this, the evaporator with a hydrophobic photothermal layer on its upper surface and a hydrophilic layer on its lower surface was called a Janus evaporator. The Janus evaporator had a positive impact on long-term desalination efficiency through its unique design features. On the one hand, the top layer of the Janus evaporator served as a hydrophobic solar absorber, which was waterproof and salt-resistant, effectively preventing salt accumulation at the evaporation interface, thereby maintaining the cleanliness of the interface and an efficient evaporation ability. This characteristic is particularly important in long-term desalination processes, as it reduces the decrease in evaporation efficiency caused by salt accumulation. On the other hand, the bottom layer of the Janus evaporator was hydrophilic, allowing for rapid water replenishment while maintaining excellent insulation. This ensured that the evaporation process could continue efficiently without reducing the desalination efficiency due to insufficient water supply or heat loss.

In practice, the evaporator would be hit by waves and swallowed in the sea because of the tough marine environment. Therefore, the CNTs@PET-P membrane needed to be stable to resist unexpected external environments. We sonicated the CNTs@PET-P membranes in sulfuric acid, sodium chloride, and sodium hydroxide solutions for half an hour, simulating acidic, salt, and alkaline environments, and set deionized water as the control experiment. As shown in Figure 4b, no CNT detachment was observed in any of the four beakers. This further confirmed that the CNTs@rPET-P membrane could resist harsh environments. In order to give the membrane a stable folding structure, we also studied its wrinkling properties.

As shown in Figure 4c, the initial CNTs@rPET-P membrane was smooth, without any creases. After folding once, the creases were already very obvious, which proved that the as-spun CNTs@rPET membrane had an excellent wrinkle stability, which could then be folded into the designed structure. The experimental design is shown in Figure 2.

### 3.3. Evaporation Performance of CNTs@rPET-P Membranes

**Figure 5:**
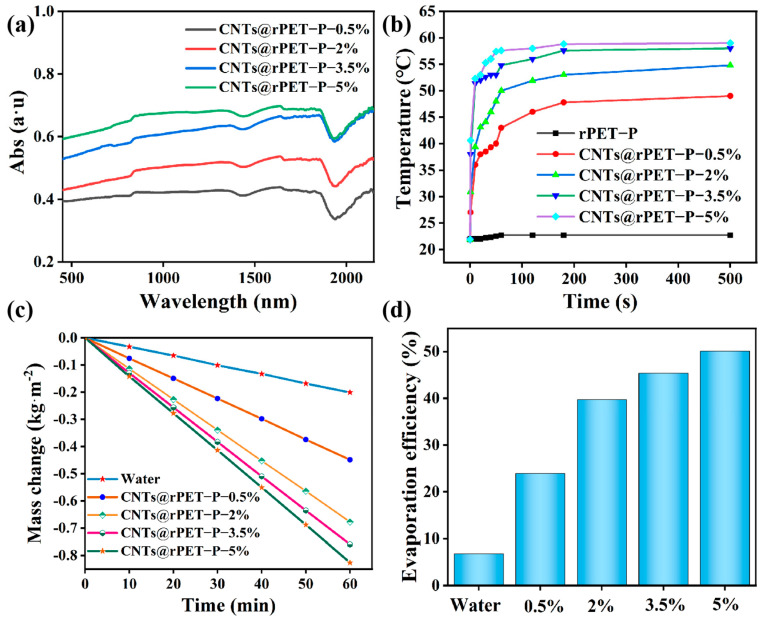
(**a**) UV–Vis–NIR absorption spectra. (**b**) Surface temperature change in dry state. (**c**) Water mass change without and with CNTs@rPET-P films. (**d**) Evaporation efficiency of CNTs@PET-P.

The light absorbance of the prepared CNTs@rPET-P membranes was measured in the 350–2500 nm spectral range. Nearly the whole spectrum was covered by the film’s absorption broad, as seen in Figure 5a, and its capacity to absorb light increased as the CNT concentration increased. The CNTs@rPET-P membranes could quickly transform light energy into thermal energy due to their superior light absorption capabilities. As suggested in Figure 5b, the solar-driven temperature of the CNTs@rPET-P membranes in the dry state rapidly increased within 60 s and reached equilibrium within about 100 s. In particular, the CNTs@rPET-P-3.5% and CNTs@rPET-P-5% membranes reached 58.0 °C and 59.0 °C, respectively. However, the membrane without CNTs had the lowest temperature, basically remaining at around 24 °C.

Steam can be evaporated under heat energy transformed from light energy. We also evaluated the seawater evaporation ability of the CNTs@rPET-P evaporators with different CNT concentrations under a single solar intensity (1 kw/m^2^), repeating this five times. Firstly, we put the evaporator and a beaker of seawater onto an electronic balance, and then recorded the seawater mass change. It was found that the water mass loss of the CNTs@rPET-P-3.5% and CNTs@rPET-P-5% evaporators within 1 min was 0.76 and 0.827 kg·m^−2^, respectively, much higher than the mass loss of pure water nature evaporation (0.201 kg·m^−2^), as displayed in Figure 5c. However, when CNTs reach a certain concentration, CNT particles will aggregate together, thereby affecting the photothermal conversion effect. Therefore, there was not much difference in the water quality loss between the CNTs@rPET-P-3.5% and CNTs@rPET-P-5% evaporators. The evaporation efficiency exhibited the same pattern as well. Figure 5d illustrates how the evaporation efficiency rose as the concentration of carbon nanotubes did. The seawater natural evaporation efficiency was only 7.2% while the evaporation efficiencies of the CNTs@rPET-P-3.5% and CNTs@rPET-P-5% evaporators were 45.4% and 50.1%, showing that the evaporators functioned well in terms of water evaporation.

### 3.4. Optical Property of F@N

**Figure 6:**
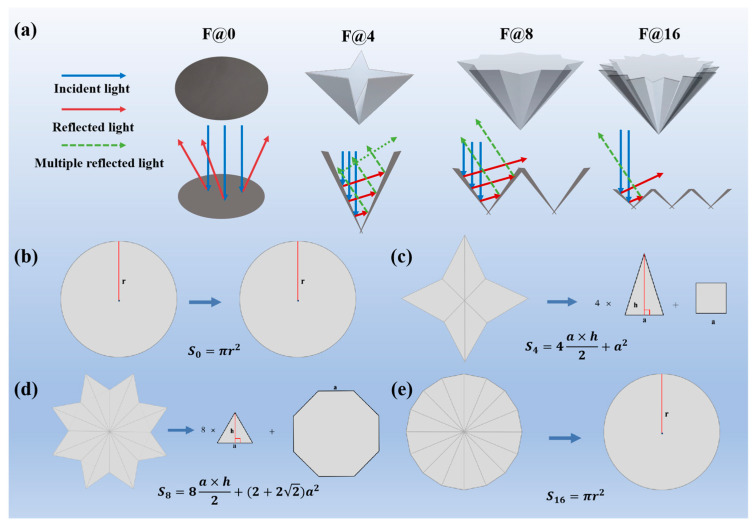
(**a**) The structure diagram of F@0, F@4, F@8, and F@16 and the interaction diagram of incident light. (**b**–**e**) The projection area calculation of F@0, F@4, F@8, and F@16.

To find the effect of the folded structure on the photothermal property, we folded CNTs@rPET-P-5% membranes with different numbers of folded petals, named F@0 (planar structure), F@4, F@8, and F@16, respectively. A greater folding number would destroy the double-layer structure of the CNTs@rPET-P-5% membrane, making the CNTs@rPET nanofibrous films fall off the air-laid paper. As illustrated in Figure 6a, the folding structure enhanced light absorption by increasing the quantity of light reflections. Once incident light entered the folded petals, the reflected light could hit the inside walls again and then cause a multi reflection and absorption [28,34]. It was evident that the height of the petals steadily decreased as the number of folded petals increased, along with the number of incident light reflection gradually decreasing, which might result in the absorption capacity of light gradually weakening.

Since the evaporation performance of the evaporator depended on the light intensity and irradiation area, the area we used for calculation referred to the projected area. With a constant light intensity and the same irradiation time, the smaller the projected area, the higher the efficiency obtained. Here, we used the same initial surface area for different folding structures, but the projected area after folding was different. Therefore, we defined a specific surface area of F@N through dividing the surface area by the projected area as an indicator of its evaporation performance. Firstly, after folding a circle with a radius of 2.5 cm into different structures, the projected areas of F@0, F@4, F@8, and F@16 were calculated separately, which can be visually observed in Figure 6b–e.

(1) $$S_{0}=\pi r^{2}$$

Among them, $S_{0}$ is the projected area of F@0 and *r* is the radius of the circle in Figure 6b, (*r* = 2.5 cm). After calculation with π=3.14, $S_{0}$ = 19.625 cm^2^.

(2) $$S_{4}=4\frac{a\times h}{2}+a^{2}$$

Among them, $S_{4}$ is the projected area of F@4, *a* is the length of the base of the triangle and the side length of the square in Figure 6c, and *h* is the height of the triangle (*a* = 2 cm and *h* = 1.6 cm). After calculation, $S_{4}$ = 10.4 cm^2^.

(3) $$S_{8}=8\frac{a\times h}{2}+(2+2\sqrt{2})a^{2}$$

Among them, $S_{8}$ is the projected area of F@8, *a* is the length of the base of the triangle and the side length of the regular octagon in Figure 6d, and *h* is the height of the triangle (*a* = 1 cm and *h* = 0.8 cm). After calculation, $S_{8\;}$ = 8 cm^2^.

(4) $$S_{16}=\pi r^{2}$$

Among them, $S_{16}$ is the projected area of F@16, *r* is the radius of the circle in Figure 6e, (*r* = 2 cm), and the reduction in the radius was due to the folding. After calculation with π=3.14, $S_{16}$ = 12.56 cm^2^.

Subsequently, we divided the surface area by the projected area to define the specific surface area of F@N as an indicator of its evaporation performance.

(5) $$S_{ssa}=\frac{S_{sa}}{S_{N}}$$

(6) $$S_{sa}=2\pi r^{2},(r=2.5\mathrm{cm})$$

Among them, $S_{ssa}$ is the specific surface area, $S_{sa}$ is the total surface area of the unfolded membranes with front and back side surfaces, and $S_{N}$ is the projected area (*N* = 4, 8, and 16). From Figure 2, it can be seen that the surface areas of F@0, F@4, F@8, and F@16 were the same ($S_{sa}=3$9.25 cm^2^).

**Figure 7:**
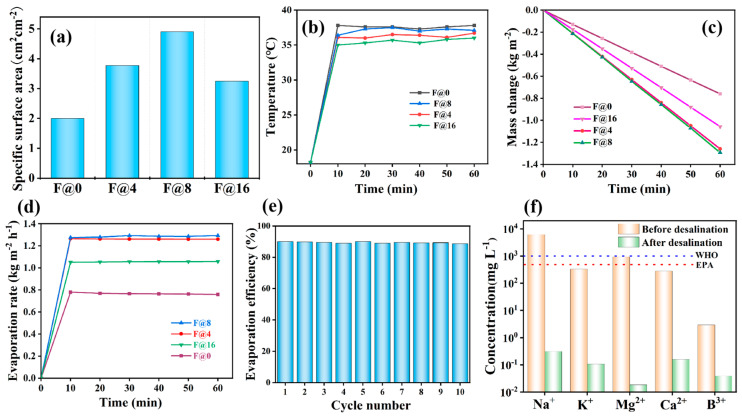
(**a**) Specific surface area of F@N evaporator, (**b**) temperature of working F@N evaporator varies with time, (**c**) F@N evaporator induced seawater mass change with time, (**d**) evaporation rate of the designed F@N evaporator within 1 h, (**e**) evaporation efficiency of F@8 within 10 cycles, and (**f**) ions changes before and after desalination with F@8 evaporator.

According to calculations, the projected areas of F@0, F@4, F@8, and F@16 were 19.625, 10.4, 8, and 12.56 cm^2^. According to the specific surface area calculation formula in (5), the specific surface areas of F@0, F@4, F@8, and F@16 were 2, 3.774, 4.906, and 3.125 cm^2^·cm^−2^. The specific area can be used to explain the laws of change in evaporation efficiency. It was noted that, as the number of folded petals increased, the specific surface area first improved and then decreased with cross peaking at F@8, as suggested in Figure 7a. This might have been because the increase in folded petals made the projection area of F@N decrease first and then increase, and the projection shape of F@16 was close to the circle, reaching the maximum.

### 3.5. Photothermal Performances of F@N

The F@N evaporator’s surface temperature change during water evaporation is depicted in Figure 7b. Generally, the surface temperature of F@0 was higher than that of the folded ones. Because the water evaporator was moist during operation, water evaporation required heat absorption. The folding structure itself evaporated more water than the flat one, so the temperature of the folding evaporators was lower than that of the flat one. For the folded evaporators, the surface temperature of F@8 was highest. This was due to the F@8 evaporator having the highest light absorption specific area, so more incident light was absorbed and more heat was converted.

The F@N evaporator’s water evaporation capabilities are depicted in Figure 7c,d. It was found that all the folded evaporators had greater evaporation rates and water mass changes than the F@0 evaporator. This resulted from the fact that the folded structure had greater light absorption and then converted more heat into more water evaporation. In addition, the nanofibrous surface of the membrane was conducive to improving the evaporation area of water, providing more ways for steam to escape. For the folding evaporators, F@4 and F@8 had respective evaporation rates of 1.29 and 1.31 kg·m^−2^·h^−1^, while F@16 had an evaporation rate of about 1.05 kg·m^−2^·h^−1^. It was indicated that the folding number was not the key point for the evaporation rate, but the light absorption specific area was a critical factor.

The performance stability of an evaporator is the standard to ensure whether it can work continuously for a long period of time. In order to examine the long-term performance stability of the designed folded evaporator, the F@8 evaporator was tested for 10 cycles (60 min each time in 10 days). It was found that the F@8 evaporator could maintain a stable high photothermal performance with about a 90.59% evaporation efficiency (Figure 7e). We also checked the quality of the desalination water by checking the concentrations of ions, including Na^+^, K^+^, Ca^2+^, Mg^2+^, and B^3+^. After seawater desalination, the ion concentration was greatly lowered by two to three orders of magnitude, as illustrated in Figure 7f, and the level completely satisfied the WHO and EPA drinking water quality criteria.

### 3.6. Cost Benefit Analysis of F@N

With the deepening of research, blindly improving photothermal conversion efficiency would undoubtedly increase the complexity and cost of the preparation process. Here, we used the “photothermal quality factor” as a comprehensive indicator for evaluating the photothermal performance of evaporators [32]. The calculation formula for quality factor Q is as follows:

(7) $$Q=\frac{\ln\left(\eta \right)}{C}$$

where Q is the quality factor, m^2^/$; η is the photothermal conversion efficiency,%; and C is the cost, USD/m^2^. The logarithmic function of efficiency did not change the nature or correlation of efficiency, but could reduce the scale of variables, making the correlation between the quality factor and cost more prominent. The larger the Q value, the better the overall performance of the designed photothermal evaporator.

Table 1 lists the materials, evaporation rate, cost, and quality factor of the F@8 evaporator, and compares it with other relevant research. From this, it can be seen that a high efficiency and low cost were the factors for obtaining high-quality factors. This study conducted experimental research using rPET and carbon nanotubes as raw materials, with a quality factor of up to 17.33. It can be seen that, compared with other studies, the evaporator prepared in this article has significant advantages in terms of cost-effectiveness.

## 4. Conclusions

In summary, we designed a folded structure 3D evaporator based on electrospun rPET membranes with CNTs as light absorption materials. Through comparative experiments, it was found that the evaporator with a CNT concentration of 5 wt% had the best evaporation performance. Subsequently, through continuous folding of the photothermal membranes with 4, 8, and 16 petals, it was found that F@8 (with 8 folded petals) had the best photothermal performance, with a water evaporation rate of 1.31 kg·m^−2^·h^−1^, maintaining a high photothermal conversion rate of approximately 90.59% after 10 cycles. The results indicate that there was an optimal number of folded petals, which mainly depended on the final light absorption area. This approach would provide a new and costly method for evaporator design. The folding structure may not only be suitable for interfacial evaporators, but could also benefit reverse osmosis desalination.

## Figures and Tables

**Table 1:** Comparison of photothermal performance, material cost, and quality factor under a single sun (1 kw/m^2^).

Literature	Structure	Material	Evaporation Efficiency (%)	Cost (USD/m^2^)	Quality Factor (m^2^/$)
F@8	Eight folded petals	CNTs, rPET, air-laid paper	90.59	0.26	17.33
[37]	Cone-shaped array	Tannic acid, ferric chloride, 3D printing ink	94.4	≈11	0.41
[38]	Three-dimensional porous cubic shape	Silver nitrate, polyvinylpyrrolidone, silica, squid powder	94	≈2	2.27
[39]	Porous cylinder	Lanthanum nitrate hexahydrate, cobalt nitrate hexahydrate, strontium nitrate, polyvinyl alcohol solution, chitosan solution	93	≈3	1.51
[40]	Durian shell shaped	Pyrrole, iron oride hexahydrate	91	≈1	4.51

## Data Availability

The raw data supporting the conclusions of this article will be made available by the authors on request.
